# Fade into you: genetic control of pigmentation patterns in red-flesh apple (*Malus domestica*)

**DOI:** 10.3389/fpls.2024.1462545

**Published:** 2025-01-13

**Authors:** Pierre Bouillon, Etienne Belin, Anne-Laure Fanciullino, Sandrine Balzergue, Sylvain Hanteville, Yao Letekoma, Maryline Cournol, Fatima Faris, Andréa Bouanich, Dimitri Bréard, Frédéric Bernard, Jean-Marc Celton

**Affiliations:** ^1^ Univ Angers, Institut Agro, INRAE, IRHS, SFR QUASAV, Angers, France; ^2^ IFO, Seiches sur le Loir, France; ^3^ Analyses des Acides Nucléiques (ANAN), SFR QUASAV, Angers, France; ^4^ Univ Angers, Substances d’Origine Naturelle et Analogues Structuraux (SONAS), SFR QUASAV, Angers, France

**Keywords:** red-flesh apple, *Malus domestica*, anthocyanin, pigmentation pattern, apple genetics

## Abstract

The genetic basis of type 1 red-flesh color development in apple (*Malus domestica*) depends upon a particular allele of the *MdMYB10* gene. Interestingly, type 1 red-flesh apples are fully red after fruit set, but anthocyanin pigmentation in apple fruit cortex may decrease during fruit growth and maturation, leading to variable red patterning and intensities in the mature cortical flesh. We developed a histogram-based color analysis method to quantitatively estimate pigmentation patterns. This methodology was applied to investigate the phenotypic diversity in four hybrid F1 families segregating for red-flesh color. Pigmentation patterns were found to be heritable allowing the identification of a new locus by QTL analysis. To further investigate the mechanisms involved in the spatial deposition of anthocyanin, metabolome, transcriptome and methylome comparisons between white and red flesh areas within the red-flesh genotype cv. ‘R201’ exhibiting flesh pigmentation patterns, was performed. Wide-targeted analysis showed that white-flesh areas accumulate more dihydrochalcones and hydroxycinnamic acids than red-flesh areas while red-flesh areas accumulate more flavonoids. Anthocyanin biosynthesis genes and anthocyanin positive regulators (MBW complex) were up-regulated in red-flesh areas, while a reduction in anthocyanin storage, transport and stability (increase of pH, down-regulation of *MdGSTU22*) and an increase in phenolic catabolism were concomitant with color fading process in white-flesh areas. Expression of *MdGSTU22* was linked to a differentially methylated region (DMR) suggesting a potential environmental effect on the epigenetic control of gene expression involved in color fading. Altogether, these results provide the first characterization and functional identification of color fading in apple fruit flesh.

## 1 Introduction

Plant pigments have long fascinated scientists who have studied their chemical and physical properties, their biosynthesis, and their physiological and ecological functions (Grotewold, 2006). Anthocyanins, a class of flavonoids, are water-soluble phenolic compounds and pigments that provide vibrant hues in the plant kingdom, from orange/red to violet/blue (Tanaka et al., 2008). The sequential steps leading to biosynthesis of anthocyanins have been well described and are highly conserved among species (Buhrman et al., 2022). Anthocyanins synthesis begins with the condensation of three molecules of malonyl-CoA and one of p-coumaroyl-CoA from phenylalanine via the general phenylpropanoid pathway. Malonyl-CoA and p-coumaroyl-CoA are condensed by a chalcone synthase (CHS). Four enzymes in flavonoid biosynthesis, chalcone isomerase (CHI), flavanone 3-hydroxylase (F3H), dihydroflavonol 4-reductase (DFR) and anthocyanidin synthase (ANS or LDOX), are then required to achieve anthocyanidin synthesis (Zhang et al., 2014). Stable anthocyanins are then glycosylated on the 3-position by a (UDP)-glucose:flavonoid 3-O-glucosyltransferase (UFGT) (Liu et al., 2021).

Following primary synthesis, anthocyanins are transported to the vacuole where they accumulate and participate in the final color acquisition. Glutathione S-transferases (GSTs) (Zhao et al., 2021b), ATP-binding cassette (ABC-transporters) (Xiang et al., 2024) and toxic compound extrusion MATE-transporters (Zhang et al., 2021) are key enzymes that enable anthocyanin translocation from cytoplasm into the vacuole (Buhrman et al., 2022). In peach fruit (*Prunus persica*), loss-of-function alleles of the glutathione S-transferase (GST) gene causes anthocyanin deficiency in flower and fruit skin (Lu et al., 2021). In apple, *MdGSTU12* was shown to play an important role in the regulation of anthocyanin accumulation in fruit skin (Zhao et al., 2021b). In kiwifruit, methylation of AcGST1 is associated with differential accumulation between the inner and outer pericarp (Zhang et al., 2023). Indeed, a decrease in the methylation level of the *AcGST1* promoter may contribute to accumulation of anthocyanin in the outer pericarp of the fruit.

In addition to structural genes involved in anthocyanin biosynthetic pathway, several transcription factors (TFs) have been found to regulate anthocyanin accumulation (Allan et al., 2008). The ternary MBW protein complex comprising MYB, bHLH, and WD40 proteins is considered to be a major transcriptional regulator of anthocyanin synthesis (Chen et al., 2021). R2R3-MYB activators can bind, directly or within the MBW complex, to the promoters of anthocyanin biosynthetic genes (ABGs), thereby increasing their expression and modulating subsequent anthocyanin accumulation (Yan et al., 2021). In contrast, a large number of anthocyanin repressors have been identified in the last decade (LaFountain and Yuan, 2021). Some R2R3-MYB and R3 repressors are generally involved in interfering with MBW complex to inhibit activation of the anthocyanin regulatory network (LaFountain and Yuan, 2021), and activator–repressor MYB pairs have been found to control the formation of heterogeneous pigmentation region in many angiosperm species (Davies et al., 2012).

The color fading process, described as the loss of anthocyanin pigmentation in plant tissues and organs at particular developmental stages, is well-known in ornamental species, particularly in flowers (Han et al., 2020; Huang et al., 2024). While regulatory mechanisms of anthocyanin biosynthesis are well described in many plant species, the genetic and biochemical mechanisms that lead to anthocyanin degradation are less well understood. In *Malus halliana*, repression of *MhMYB10* was associated with flower color fading (Han et al., 2020), suggesting that the increased methylation level of MYB10 slowed the anthocyanin accumulation process by repressing its expression. The importance of micro-RNAs (miRNAs) in anthocyanin regulation has also been characterized in *Malus Crabapple* where numerous miRNAs have been linked to petal color fading (Rong et al., 2023). In pear leaves and flowers, overexpression of a laccase through MYB TF activation resulted in color fading (Zhao et al., 2021a).

Study of pigmentation patterning is an exciting area (Fairnie et al., 2022) that can be traced back to the Reaction-Diffusion model established by Alan Turing (Kondo and Miura, 2010) that finds echoes in recent genetic models proposed on Monkeyflower (*Mimulus*) (Yuan, 2018; Ding et al., 2020). Color patterning can be dependant upon an activator-repressor system involving in the upregulation of an inhibitor during later steps of tissue development. For example, an activator-repressor system was observed in red-flesh kiwifruit (*Actinidia* spp.) where an interplay between positive regulation of MYB TF and negative regulation by mircoRNAs (miRNAs) regulated anthocyanin synthesis and distribution (Wang et al., 2022). An activator-repressor system was also identified in peach where a R2R3-MYB repressor, PpMYB18, competes with PpMYB10 for binding to bHLH in a fine-tuning regulatory loop to balance anthocyanin accumulation (Zhou et al., 2018). In contrast, traits without repeating pattern are generally explained in terms of positional specification, in which overlapping zones of gene expression regulate pigmentation pattern (Zheng et al., 2021). While biological functions of flower pigmentation pattern are well-studied in pollinators attraction (Moyers et al., 2017; Todesco et al., 2022) and defense against herbivore (Lev-Yadun, 2016), the biological process that leads to pigmentation pattern in fruits are much less understood. Recent progress in image analysis opens up a new field of study for pigmentation pattern with recent applications in fruit species such as strawberry (*Fragaria × ananassa*) (Zingaretti et al., 2021; Denoyes et al., 2023). In apple, fruit color is associated with anthocyanin accumulation (Allan et al., 2008). The major apple phenolic compounds are flavonols, monomeric and oligomeric flavan-3-ols, dihydrochalcones, hydroxycinnamic acids and anthocyanins, with cyanidin-3-galactoside accounting for about 80% of the total amount of anthocyanins in apple (Chen et al., 2021). Some genotypes display red pigmentation in the fleshy part of the fruit leading to the ‘red-flesh’ trait. Two different types of red-flesh apples have been characterized. The type 1 red-flesh phenotype accumulates anthocyanins throughout the fruit, from fruit set through maturity and is associated with red pigmentation of leaves, stems, roots, and flowers. The type 2 red-flesh apple phenotype is characterized by green vegetative tissues, yellow-orange fruit skin, and a red pigmentation that occurs only in the fruit flesh during later stages of fruit development (Chagné et al., 2012). Type 1 red-flesh is dependent upon the presence of a particular allele of the *MdMYB10* gene (historically termed “R” locus) (Chagné et al., 2007; Espley et al., 2007). This gene, characterized by a minisatellite-like structure, is required for red-flesh pigmentation by upregulating anthocyanin late biosynthetic genes (Espley et al., 2009). Other genetic factors have been identified through recent genetic analyses (Kumar et al., 2022). Transcriptomic analyses identified differentially expressed genes (DEGs) associated with the flavonoid pathway between red-flesh and white-flesh apples (Wang et al., 2015). Among them, the WRKY-family TF *MdWRKY11* seems to be a major regulator of flavonoid and anthocyanin synthesis (Wang et al., 2018), and is involved in vacuolar acidity (Verweij et al., 2016; Lloyd et al., 2017). Recently, Li et al (Li et al., 2022), observed that ABGs were more abundantly expressed in the red areas of the fruit than in the white sections. They identified, through transcriptomic analysis, DEGs related to phytohormones and involved in anthocyanin biosynthesis regulation, and proposed that some auxin and ethylene response TFs could participate in anthocyanin biosynthesis. *MdNAC42* was observed to interact with *MdMYB10* in red-flesh apple, and up-regulated the expression of several apple ABGs, leading to an increased fruit coloration (Zhang et al., 2020). *MdMYB9* also participates in anthocyanin and proanthocyanidin synthesis through a HY5-miR858-MYB loop (Li et al., 2023). MYB repressors of anthocyanin synthesis have been successfully identified in apple. *MrMYB44* caused color fading of *Malus radiant* leaves (Meng et al., 2022), and was closely linked to fruit malate accumulation (Jia et al., 2021). Interestingly, in strawberry, *FaMYB44* was found to interact with *FaMYB10* during development and regulate sugar/acid accumulation (Wei et al., 2018). In type 1 red-flesh apple, *MdMYB6*, *MdMYB16*, *MdMYB17* and *MdMYB111* are responsible for downregulation of anthocyanin structural genes (Xu et al., 2020; Wang and Chen, 2021).

Red-flesh fruits are fully red after fruit set (Espley et al., 2012), however, it has been observed that anthocyanin pigmentation in apple fruit cortex may decrease during the course of fruit growth and maturation, leading to variable red patterning and intensities in the cortical flesh (van Nocker et al., 2011; Volz et al., 2014; Bouillon et al., 2024a). This study aimed to identify genomic regions associated with flesh pigmentation pattern in apple. Red-flesh pigmentation pattern was found to be heritable in a hybrid progeny, underlying a potential genetic control of this trait (Bouillon et al., 2024a). In this study, a QTL analysis approach was combined with a differential transcriptome and a methylome analysis to decipher the genetic basis of pigmentation pattern in red-flesh apple fruit. A pedigree-based QTL analysis (PBA-QTL) was conducted using four F1 red-flesh apple progenies (256 individuals), supplemented with genetic data from parents, grandparents and founders (totaling 347 individuals) to characterize the genetic architecture of flesh pigmentation pattern. To conduct this analysis, an innovative phenotyping method was developed and implemented to quantitatively estimate flesh pigmentation pattern in red-flesh fruits. Comparison between inner white-flesh (WF) and outer red-flesh (RF) metabolome, transcriptome and methylome of a red-flesh cultivar (cv. *‘R201’*) that exhibited flesh pigmentation pattern was then performed.

## 2 Material and methods

### 2.1 Plant material

A pedigree-based QTL analysis (PBA-QTL) was performed on 256 individuals from four interconnected (common parent and/or grandparent) F1 families (Bouillon et al., 2024a), supplemented with genetic data from parents, grandparents and founders (totaling 347 individuals). Three families segregated for red-flesh (RF1-1 to RF1-3, plantation year: 2018) and one family was a cross between two red-flesh parents (RF1-5, plantation year: 2017). Hybrids were selected at the seedling stage for red-leaves color [phenotypic marker of *MdMYB10* (Chagné et al., 2007)] and, consequently, possessed at least one *R6-MdMYB10* allele. Fruit harvest was conducted for three years (2021, 2022 and 2023) from August to end-October in IFO orchard (L’Anguicherie, 49140 Seiches-sur-le-Loir, France/GCS: 47°37’52.5”N 0°19’38.4”W). For each genotype harvested at maturity (brix values varying from 13 to 22; starch index between 6 and 8), four representative fruits were used for image analysis. Fruits that were positioned in the middle of the tree, at similar exposure to light, same developmental stage, and similar diameter were picked preferentially to limit intra- and inter-tree bias.

### 2.2 Estimating pigmentation pattern in red-flesh apple

#### 2.2.1 Image analysis

**Figure 1:**
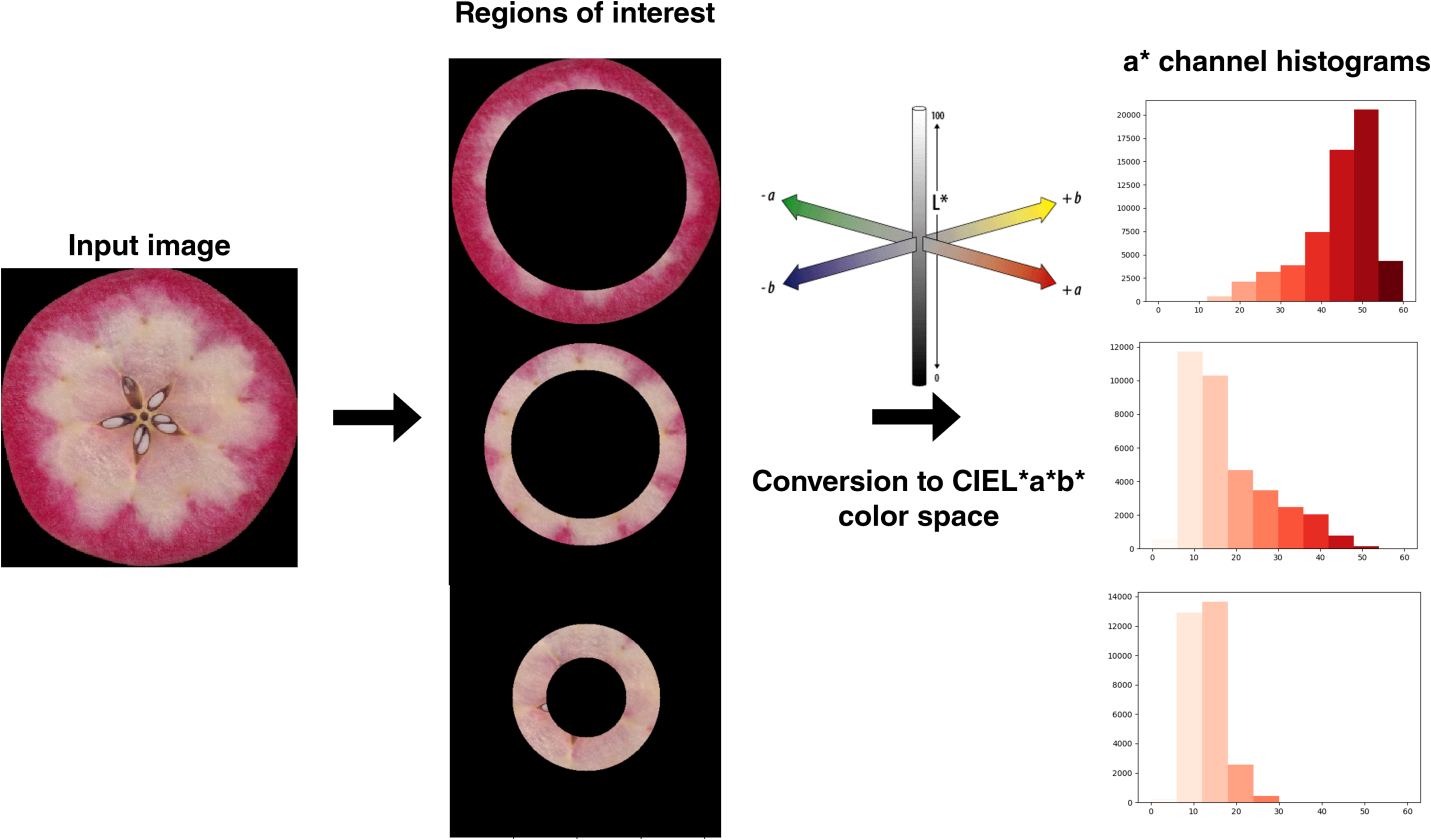
Overview of the histogram-based color analysis pipeline. RGB images of individual transversal apple sections were converted to gray images in order to establish three circular regions of interest (r.o.i). To be insensitive to size fruit variations, the three r.o.i of the fruit were defined as the ‘inner part’ (30-55% of the fruit radius), the ‘medium part’ (55-75%) and the outer part (75-100%). Radius of fruit was evaluated according to the detection of centroid and edge of each fruit. These circular ring masks were converted from RGB to CIEL*a*b* color space. From a* channel, normalized histograms with 10 fixed equal intervals (bins) between 0 and 60 on the a* values were then extracted for the 3 circular rings.

Image acquisition, segmentation and analysis was performed as described in (Bouillon et al., 2024a). Concomitantly, a histogram-based color analysis pipeline was written in Python (**Figure 1**). Color analysis was inspired from a Matlab pipeline developed to study color patterning in foliar coleus (Li et al., 2021) and ColourQuant protocol (Li et al., 2022). RGB images of individual transversal apple sections were converted to gray images in order to establish three circular regions of interest (r.o.i). To be insensitive to size fruit variations, the three r.o.i of the fruit were defined as the ‘inner part’ (30-55% of the fruit radius), the ‘medium part’ (55-75%) and the outer part (75-100%). We excluded carpels (0-30%) to avoid bias in color estimation of fleshy tissue. Radius of fruits was evaluated according to the detection of centroid and edge of each fruit, from the *‘measure.regionprops’* module from scikit-image (Van der Walt et al., 2014). These circular ring masks were converted from RGB to CIEL*a*b* with Scikit-image *rgb2lab* function. CIEL*a*b* is a device-independent continuous color space that consists of three channels L* = “lightness”, a* = “green to magenta”, b* = “blue to yellow”. CIEL*a*b* color space enables extraction of quantitative color distribution information (Li et al., 2021). Color variations in apple fruits are constrained from green to red, so we chose the a* channel as an indicator of flesh color variations (Bouillon et al., 2024a). Normalized histograms with 10 fixed equal intervals (bins) between 0 and 60 on the a* values were then extracted for the 3 circular rings.

#### 2.2.2 Phenotypic data

Initial included 4933 images x 30 bins (10 bins for 3 circular rings) representing 492 individuals for three years (2021-2022-2023). Principal component analysis was performed on the color dataset. Correlations between coordinates of PCs and color descriptors were calculated to identify components associated with color heterogeneity/pigmentation pattern (Bouillon et al., 2024a). Each genotype was expressed as mean of its 4 fruits coordinates each year. A previous QTL analysis identified a QTL on LG16 linked to mean of a* values that colocalized with a QTL identified in a red-flesh biparental population (Bouillon et al., 2024b). In order to exclude low-colored genotypes that could display positive alleles for pigmentation pattern, thus rendering phenotyping very challenging, a subset of genotypes were filtered for mean of a* values (> 10) and the presence of LG16-QTL “F” allele at haploblock HB-16-5 (approximately 8 cM). Following this selection, 256 F1 red-flesh genotypes remained for QTL detection (**Supplementary Table S1**).

### 2.3 QTL analysis

#### 2.3.1 Statistical analysis

Best linear unbiased prediction (BLUP) was used to estimate random effects of a mixed linear model (Piepho et al., 2007) with the following formula:


y=Xβ+Zu+e

where y is the vector of observations (phenotypic scores), *β* and u are vectors of fixed (year effect) and random effects (genotypic values), respectively, X and Z are the associated design matrices, and **e** is a random residual vector. The random effects are estimated by BLUP (Piepho et al., 2007). Because of our experimental design, not all environmental effects could be considered (tree and genotype effects were confounded), and BLUPs of across-year phenotypic values per genotype approximated the true genotypic values. BLUP was estimated from scores of individuals on the second PCA component for 2021-2022-2023 image data and used for QTL detection.

Broad-sense heritability was estimated by intra-class correlation analysis (Xu, 2022) with the following formula:

${\text{h}}^{2}=\sigma_{g}^{2}/(\sigma_{g}^{2}+\sigma_{r}^{2})$ where $\sigma_{g}^{2}$ and $\sigma_{r}^{2}$ were the individual genetic and residual variances, respectively.

#### 2.3.2 Genotypic data

Hybrids, their parents and ancestors were genotyped using the Illumina Infinium 20K SNP array for Apple (Bianco et al., 2014). The raw SNP data was initially processed into the GenomeStudio software v2.0 and SNP data curation was performed as described by Vanderzande et al (Vanderzande et al., 2019). SNP physical position on the Malus genome (Daccord et al., 2017) was derived from the integrated genetic linkage (iGL) map for Illumina Infinium 20K SNP array (Howard et al., 2021). A total of 4220 informative SNP were retained. SNP markers were well distributed over the 17 chromosomes. Founders were added in our pedigree from public genotyping data on GDR (Jung et al., 2018). Thus, we were able to trace marker segregation from our genotypes to the wild red-flesh accession *Malus Sieversii f. niedzwetzkyana*.

#### 2.3.3 Haplotype determination

‘PediHaplotyper’ v.1.0 was used to construct haplotypes (Voorrips et al., 2016). Maximum size of each haploblock was limited to 1 cM. Identical haplotypes were described as IBD if they could be traced back to a common ancestor, whereas identical haplotypes that could not be traced back to a common ancestor were considered identical-by-state (IBS). Missing SNP data was imputed by examining both progenies and ancestors (Powell et al., 2022). Haplotypes that could not be resolved were excluded from further analysis. Finally, a total of a 1104 haploblocks were considered for QTL detection.

#### 2.3.4 QTL detection

QTL analyses were perfomed using the FlexQTL™ software, which implements PBA-QTL analysis via Markov Chain Monte Carlo (MCMC) simulation (www.flexqtl.nl) (Bink et al., 2014). Parameters for the analyses are reported in **Supplementary Table S2**. Analyses reached adequate MCMC convergence. The model applied using FlexQTL™ software was an additive, biallelic (Q/q) model where high phenotypic values were associated with Q and low phenotypic values were associated with q. Two replicate runs with different starting seed numbers were conducted to ensure repeatability of QTL results (Kostick and Luby, 2022; Powell et al., 2022). The Bayes Factor (BF; 2lnBF10) and posterior intensity values were used to examine significance and stability of a putative QTL with evidence of a QTL being considered positive, strong, or decisive if BF were 2 to < 5, 5 to < 10, and > 10, respectively (Kostick and Luby, 2022). QTL intervals were defined in a series of successive 2 cM “bins”, chromosomal segments that had a BF above 5 and the mode within a given QTL region were considered the most probable QTL position. The proportion of phenotypic variance explained (PVE) by a QTL was estimated by dividing the reported variance explained by the whole phenotypic variance (Verma et al., 2019).

Positive/negative alleles were defined via analyses of SNP haplotypes within QTL interval. Comparison of diplotypes (diploid combinations of two haplotypes) were used to infer haplotype effects. To determine if BLUP values were significantly different for presence versus absence of a given QTL haplotype, and were significantly different between the functional diplotypes, one-way analysis of variance (ANOVA) and Tukey’s honest significant difference (HSD) were calculated (Powell et al., 2022). To investigate candidate gene colocalization in QTL regions, genes and TFs involved in anthocyanin synthesis were searched among QTL region using the ‘GDH13.v1.1’ apple genome (Daccord et al., 2017) as reference. Genetic positions were defined as the boundaries of the two inner haploblocks surrounding the QTL region.

### 2.4 Comparative analysis between RF and WF

#### 2.4.1 Plant material

To compare RF and WF, a red-flesh parent from the population, cv. ‘R201’ (plantation year: 2013) that exhibited pigmentation pattern was chosen. Red-flesh fruit are fully red after fruit set (Espley et al., 2012). Fruit flesh pigmentation pattern develop during fruit development and is stable through maturity. To identify the biochemical and genetic factors involved in anthocyanin depigmentation, the transition stage between fully red fruits and heterogeneously pigmented fruits was targeted during fruit development (84 Days After Full Bloom - DAFB). pH was measured on red and white-flesh fruit sections using a pH-meter Seven Compact S210 (Metler Toledo) as described in (Bouillon et al., 2024a).

#### 2.4.2 Determination of phenolic contents in RF and WF

Methanol (CHROMASOLV for LC-MS), acetic acid, formic acid and dimethylsulfoxide (analytical reagent grade) were purchased from Fisher Scientific. Ultrapure water was obtained from a MilliQ advantage A10 purification system (Millipore). Hyperoside, cyanidin-3-O-galactoside chloride, cyanidin-3-O-arabinoside chloride, procyanidin B1, procyanidin B2, procyanidin C1, (+)-catechin, (-)-epicatechin, chlorogenic acid, phlorizin, avicularin and quercitrin standards were purchased from Extrasynthese. 4-p-coumaroylquinic acid was purchased from Ambinter. Reynoutrin was purchased from Clinisciences. Daidzein (internal standard) was purchased from Molekula. All standards were furnished with a certificate of analysis.

Stock solutions of procyanidin B1, procyanidin B2, procyanidin C1, phlorizin, hyperoside, avicularin, quercitrin, reynoutrin and daidzein were prepared in DMSO at a concentration of 5 mg.mL^−1^. Stock solutions of (+)-catechin and (-)-epicatechin were prepared in MeOH at a concentration of 5 mg.mL^−1^. Stock solutions of chlorogenic acid and 4-p-coumaroylquinic acid were prepared in H2O at a concentration of 5 mg.mL^−1^. Stock solution of cyanidin-3-O-galactoside and cyanidin-3-O-arabinoside chloride were prepared in MeOH containing 5% acetic acid at a concentration of 5 mg.mL^−1^. All the dilutions were then carried out in MeOH except for cyanidin-3-O-galactoside and cyanidin-3-O-arabinoside chloride in MeOH containing 5% acetic acid. Each calibration curves were set to the concentration range expected for each compound. The linearity of the calibration curves was assessed by injecting 6 levels of calibration standards in three replicates. Residuals and their distribution were monitored. LOD and LOQ were defined as 3- and 10- fold the signal-to-noise, respectively. Precision was evaluated by repeated analysis of standards within different analytical batches. Each sample were spiked with the same level of daidzein at the start of extraction procedure in order to evaluate the recovery of extraction. Data acquisition and analysis were conducted on Microsoft Excel 365® and the Minitab 19® software.

Fruits without peel were cuted and two flesh pieces were selected on the sun-exposed side of the fruit (about 5 grams per fruit). Samples were immediately frozen in liquid nitrogen, stored at -80°C and freeze-dried. This dried material was crushed into a fine and homogeneous powder and stored until analysis. Polyphenols extraction protocol used was previously described by (Bouillon et al., 2024a).

Quantitative LC-UV analysis was performed on a ThermoFisher Scientific Vanquish Flex UHPLC system equipped with a quaternary solvent manager, a sample manager and a column compartment as chromatographic part and coupled to a variable wavelength detector as detection part. A sample volume of 10 µL was injected onto a ZORBAX RRHD StableBond Aq column (2.1 x 150 mm; 80 Å; 1.7 µm). Samples were kept at 10°C and column was maintained at 30°C with a flow rate of 0.3 mL.min-1. The mobile phase consisted of 0.1% formic acid in both H2O (A) and MeOH (B) used in gradient mode as follows: from 5 to 15% B in 5 min, then 15 to 35% B from 5 to 30 min, then 35 to 50% B from 30 to 35 min, then 50 to 100% B from 35 to 37 min, hold at 100% B from 37 to 39 min, and afterward the column was re-equilibrated at initial conditions during 8 min. The UV detection was measured at different wavelengths optimized for individual compounds and are listed in **Table 1**. Data were processed using Chromeleon 7.3.2 software.

**Table 1:** Summary of validation parameters.

Compound	Retention time (min)*^a^*	UV detection max (nm)	LOD (ng)*^b^*	LOQ (ng)*^c^*	Calibration range (ng)*^d^*	R²
Procyanidin B1	12.75 ± 0.12	279	0.22	0.44	0.9 - 44.1	0.992
(+)-catechin	13.45 ± 0.34	279	0.5	1	2.5 - 99.8	0.999
Procyanidin B2	16.26 ± 0.07	279	0.25	0.5	9.9 – 496.5	1
(-)-epicatechin	16.74 ± 0.25	279	0.25	0.5	5 – 248	0.999
Chlorogenic acid	17.44 ± 0.39	325	0.49	0.99	494.5 – 4945	0.999
Cyanidin-3-O-galactoside chloride	18.62 ± 0.11	512	2.4	4.7	4.7 – 236.3	0.999
4-p-coumaroylquinic acid	20.79 ± 0.34	312	0.13	0.25	5 - 250	0.999
Procyanidin C1	21.99 ± 0.33	279	0.87	2.2	4.3 – 216.8	0.999
Cyanidin-3-O-arabinoside chloride	23.95 ± 0.12	512	2.4	4.7	4.7 - 235.3	0.998
Phloridzin	33.41 ± 0.11	284	0.09	0.23	23.1 – 692.3	0.998
Quercetin 3-O-galactoside	36.21 ± 0.17	350	0.24	0.47	0.5 - 9.5	0.999
Quercetin 3-O-xyloside	36.78 ± 0.04	350	0.1	0.24	0.2 – 7.3	0.998
Quercetin 3-O-arabinoside	37.33 ± 0.03	350	0.09	0.24	0.2 – 7.1	0.995
Quercetin 3-O-rhamnoside	37.65 ± 0.01	350	0.23	0.47	2.3 – 93.2	0.999
Daidzein (IS)	38.73 ± 0.19	250	0.1	0.25	2.5 - 49	0.996

*^a^* Values are mean ± SD (n=3); *^b^* The signal-to-noise was set to 3:1; *^c^* The signal-to-noise was set to 10:1; *^d^* Suitable for samples.

#### 2.4.3 RNAseq analysis and GO-enrichment analysis

Six samples of ‘R201’ with 3 semi-biological replicates (fruits from different trees) and distinction of cortical WF and RF were analyzed by RNA sequencing. Total RNA was extracted from fruit flesh according to the method detailed by Celton et al (Celton et al., 2014). The library construction and Pair Ends 100bp reads sequencing were completed by BGI using Illumina technology, and the quality was checked using FastQC (Andrews, 2010). Alignement, quantification and differential expression analysis were performed with ‘AnaDiff’ (Pelletier, 2022). Lists of DEGs were obtained with ‘DESeq2” (Love et al., 2014) using a p-value and BH, a method for controlling the false discovery rate (FDR), of 0.05. The control represents the WF section while the treatment is the RF outer flesh. For graphical display, colorblindness-friendly colors were provided by ‘viridis’ package.

A new functional annotation of GDDH13v1.1 (Daccord et al., 2017) was performed according to the latest proteomic Arabidposis thaliana homology. The blast tool was used with a 0.05 p-value threshold. *Arabidopsis thaliana* information were extracted from TAIR10 database (Cheng et al., 2017). Interproscan (Quevillon et al., 2005) was also used on GDDH13 v1.1 proteome. A compilation of blast results, TAIR10 database and Interproscan results enabled obtention of the new functional annotation of *Malus domestica*. From the RNAseq results, we selected differentially expressed genes with a BH *<* 0.05 and logFC > |0.5|. If logFC > 0.5, genes were considered as ‘RF overexpressed’ in comparison to WF. On the contrary, if the logFC < −0.5, genes were considered as ‘RF underexpressed’ in comparison to WF. GO enrichment was computed for ‘RF overexpressed’ and ‘RF underexpressed’ genes. These two lists of genes were treated identically but distinctly for a GO enrichment by an hypergeometric law (0.05 p-value threshold) against all the genes of the genome. GO visualizations for the three GO types (Biological Process, Molecular Function and Cellular Component) were performed with Plotly (Inc., 2015).

#### 2.4.4 Differentially methylated regions (DMRs) analysis

The same six ‘R201’ samples used for differential RNAseq analyses were used. DNA was purified according to the CTAB protocol detailed by (Porebski et al., 1997). Library construction and whole genome bisulphite sequencing (PE 100 bp) were performed by BGI TECH SOLUTIONS (Hong Kong). The quality of paired end sequencing reads (100 bp) generated from WGBS was evaluated with FastQC (version 0.11.9). The Trimmomatic (v0.39) software was used to remove adaptor sequences and poor quality bases using the given parameters “LEADING:3, TRAILING:3, MINLEN:80, ILLUMINACLIP:adapter:2:30:10”. The reference genome GDDH13 v1.1 was converted (C to T and G to A) by ‘bismark_genome_preparation’ tool within Bismark (v0.24.0) (Krueger and Andrews, 2011). Subsequently, trimmed sequencing reads were aligned to the converted genome version using bowtie2. The resulting aligned BAM files were then processed to remove duplicates with the deduplicate_bismark tool. Methylation extraction was performed on the deduplicated reads with bismark_methylation_extractor tool to generate the context-specific methylation-states (CpG, CHG and CHH) at the individual cytosine-level for each sample with the following parameters “-p –parallel 4 –bedGraph –CX_context –cytosine_report –comprehensive –gzip”. From this extraction, a genome-wide cytosine report was generated.

The R package methylkit (v1.26.0) (Akalin et al., 2012) was used to compare the methylomes between red and white fruit flesh. Pairwise comparison was conducted with two and three semi-biological replicates, respectively. Methylation levels at cytosines in CpG, CHG or CHH contexts were initially calculated using the ‘methRead’ function with mincov=4. The ‘tileMethylCounts’ function was used to summarize methylated and unmethylated base counts over tiling windows across the genome with the parameters ‘win.size=100, step.size=100, cov.bases=4’. The ‘unite’ function was used to merge and retain only bases that were covered in tiled regions of all replicates with the destrand = FALSE parameter. Subsequently, the differential methylation statistics were calculated with ‘calculateDiffMeth’ function and ‘getMethylDiff’ function was used to select the DMRs for CpG, CHG, and CHH contexts that satisfied the following parameters ‘minDiff=25 q-value=0.05’. The command ‘bedtools closest’ from Bedtools (v2.27.1) (Quinlan and Hall, 2010) with the parameter ‘-Db’ was employed on the GDDH13 annotated genes to identify and retrieve the nearest gene to each DMR (**Supplementary Table S7**).

## 3 Results

### 3.1 Principal component analysis and color descriptors relationships

**Figure 2:**
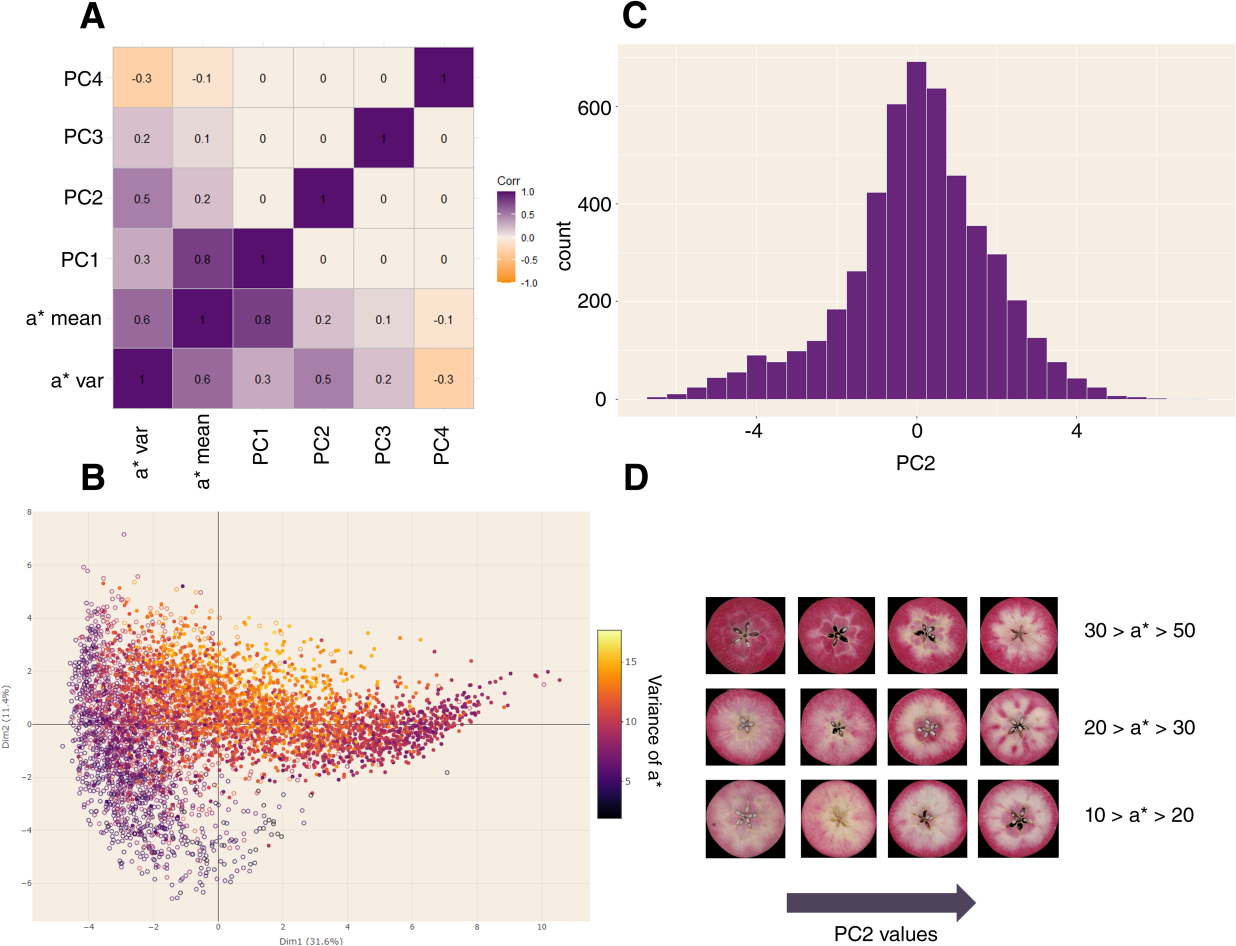
Principal Component Analysis (PCA) for the Pigmentation Patterning defined by color density based analysis. **(A)** Pearson correlation between principal components and color descriptors. **(B)** PCA plot. Genotypes are colored according to the standard deviation of a* values. **(C)** Histogram of values for the 2nd principal component (phenotypic trait of pigmentation patterning). **(D)** Visual examples of values for the 2nd principal components for different ranges of a* means.

Correlation analysis between color descriptors (Bouillon et al., 2024a) and principal components (PCs) confirmed correlation between first axis and mean of a* values (**Figure 2A**) with a Pearson correlation value of 0.8. Second axis was positively correlated to standard deviation of a* (Pearson correlation = 0.5). As expected, PC2 captured variance associated with red-flesh spatial distribution and was considered as the phenotypic trait defining pigmentation pattern (**Figure 2B**). PC1 and PC2 were not correlated, allowing decorrelation of red-flesh intensity and distribution.

Broad-sense heritability of pigmentation pattern was 0.56 and confirmed the potential genetic control of this trait. BLUP pigmentation pattern varied from -4.446 to 5.111 (**Figure 2C**) and achieved distinction between homogeneous pigmentation (white-off/yellow and pink/red homogeneous pigmented phenotypes are confounded) and pigmentation pattern (**Figure 2D**).

### 3.2 QTL detection and haplotype analysis

**Figure 3:**
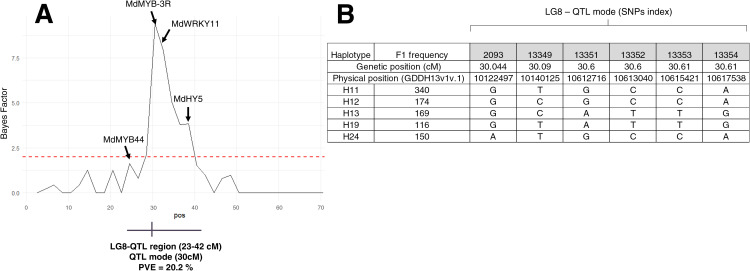
Bayes Factor (BF) accross the LG8 and genotypes for LG8-QTL haploblock **(A)** BF accross the LG8 showing significant association with pigmentation pattern. The horizontal red dashed line indicated positive threshold. The putative candidate genes underpinning the QTL region are shown. **(B)** The five haplotypes associated with LG8-QTL haploblock (HB-08-26) and their SNP sequences.

**Figure 4:**
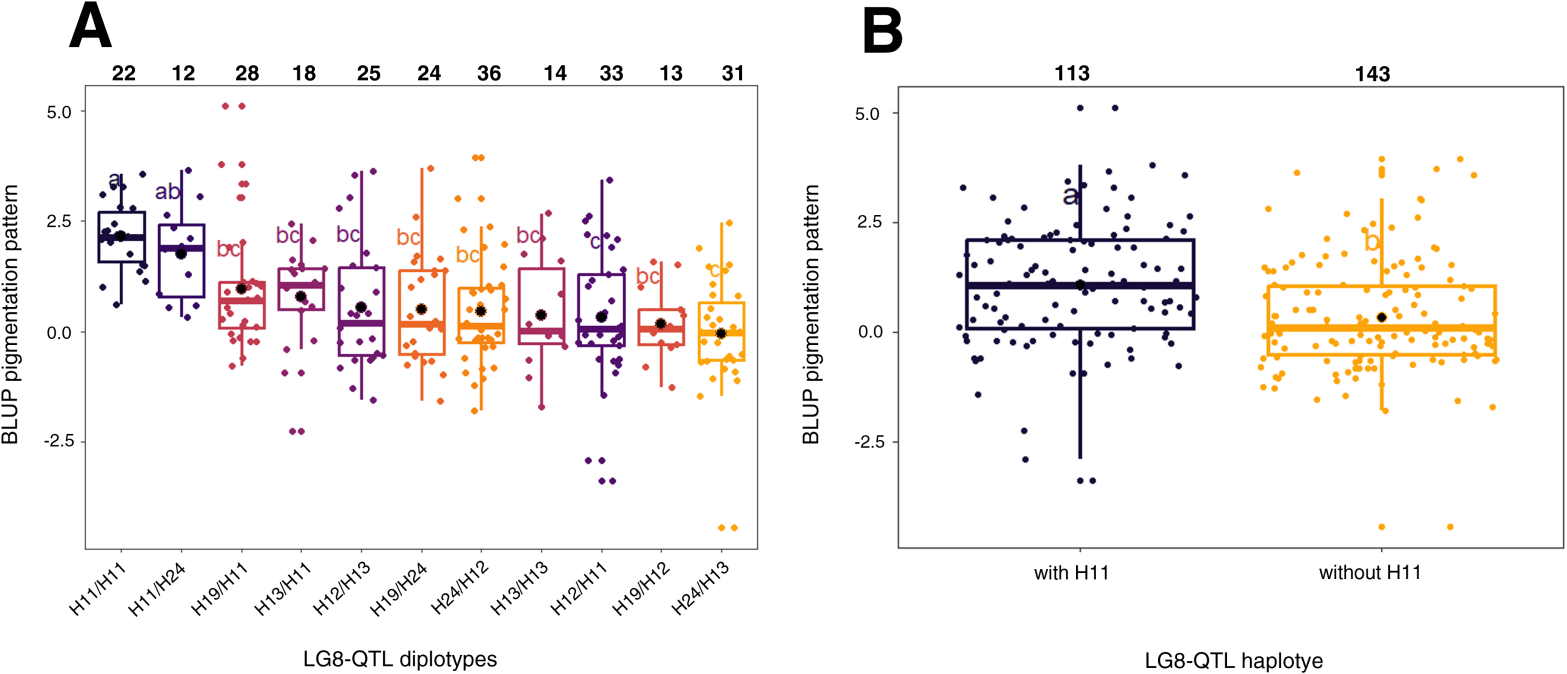
Distribution of BLUP pigmentation pattern for the five F1 offsprings haplotypes for LG8-QTL. **(A)** Distribution of the 11 diplotypes for LG8-QTL. **(B)** Distribution of the five F1 offsprings according to the absence/presence of H11 haplotype. The median (denoted by a horizontal bar in the box), the 25th percentile (denoted by the bottom edge of the box), the 75th percentile (denoted by the top edge of the box), the mean (denoted by a black dot) and the dots indicate single observations. Number of genotypes are listed above. Significantly different phenotypic means between segregating classes are identified by different letters (tukey HSD, p < 0.05).

To identify genetic regions that significantly affect pigmentation pattern, PBA-QTL analysis was performed with BLUP of pigmentation pattern used as phenotypic trait. One QTL for BLUP pigmentation pattern was detected on LG8 (**Figure 3A**; **Supplementary Table S3**). This QTL region was located between 23 and 42 cM with QTL mode located at 30 cM. Phenotypic variance explained (PVE) by this QTL was 20.2% (**Supplementary Table S4**). Haplotype analysis was conducted on LG8-QTL (**Figure 4**). Haploblock was constructed from six SNPs (**Figure 3B**) between 30.04 and 30.67 cM. Five haplotypes were identified in this population and noted H11, H12, H13, H19 and H24 (**Figure 3B**). Combination of diplotypes exhibited significant phenotypic variations on pigmentation pattern (p < 0.05; **Figure 4A**). Genotypes that displayed at least one H11 haplotype showed significantly higher phenotypic mean for pigmentation pattern (p < 0.05; **Figure 4B**). The red-flesh parent ‘R201’ was heterozygous (H19/H11) at this QTL and displayed one positive haplotype associated with pigmentation pattern.

Candidate genes associated with the LG8-QTL region were investigated (**Figure 3A**). Four putative candidate genes (among 724 genes) were identified within that region: two genes annotated for MYB (MD08G1092000 and MD08G1117700), WRKY11 (MD08G1127200) and HY5 (MD08G1147100) were located at 23, 30.6, 32.48 and 37.97 cM respectively.

### 3.3 Wide-targeted metabolite analysis between red- and white-flesh

To determine if there was significant differences in phenolic compounds accumulation between RF and WF, wide-targeted metabolite data were compared for each main phenolic groups: dihydrochalcones (phloridzin), hydroxycinnamic acids (4-p-coumaroyl quinic acid, chlorogenic acid), flavonols (quercetin 3-O-galactoside, quercetin 3-O-xyloside, quercetin 3-O-arabinoside and quercetin 3-O-rhamnoside), flavan-3-ols (catechin, epicatechin, procyanidin B1, procyanidin B2 and procyanidin C1) and anthocyanins (cyanidin 3-O-galactoside and cyanidin 3-O-pentoside).

**Figure 5:**
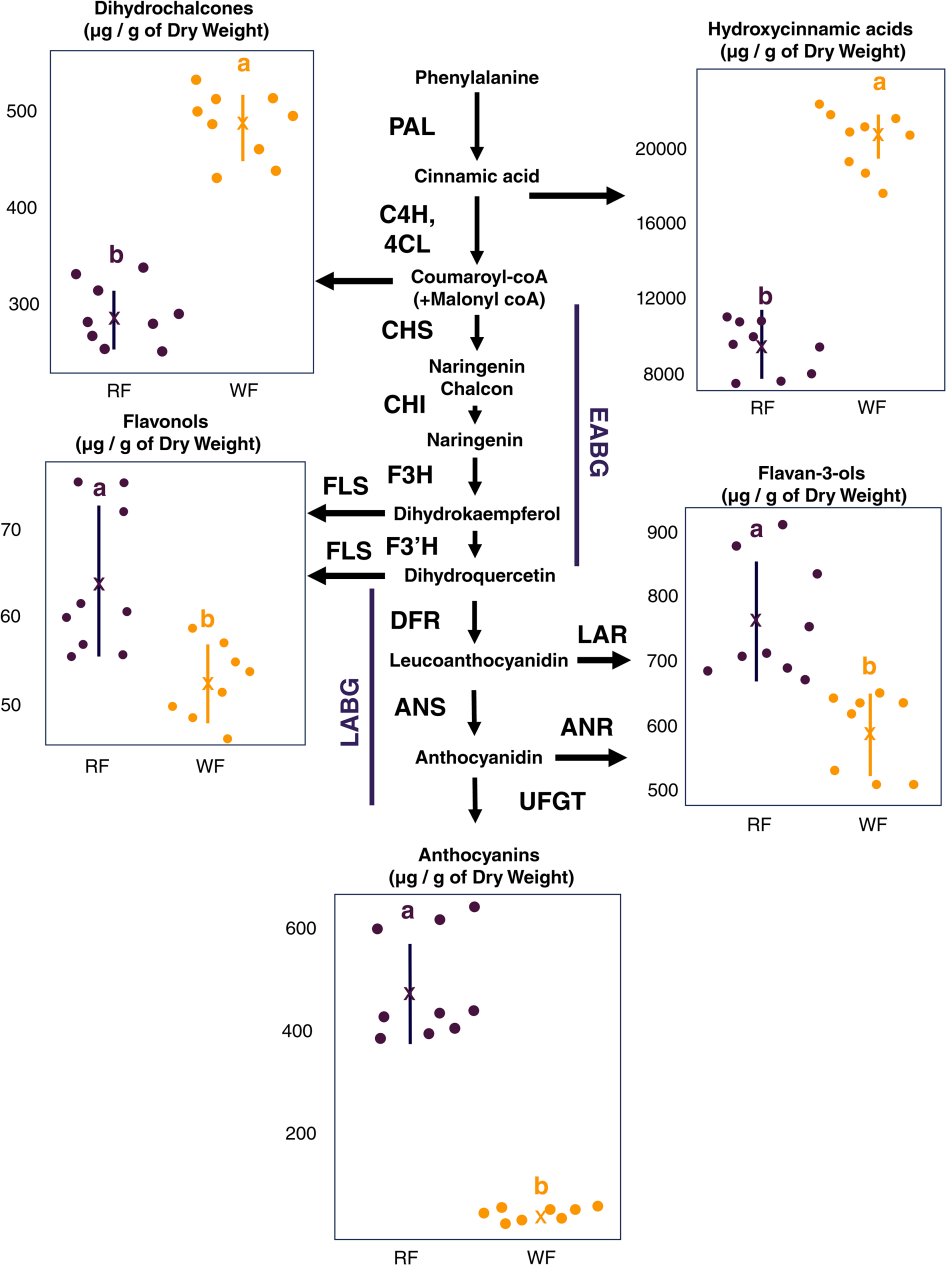
Concentration of major phenolic compounds between red-flesh (RF) and white-flesh (WF). The concentrations of these metabolites are expressed in µg.g^−1^ of dry weight. Phenolic compounds biosynthesis (enzymes and precursors) are represented. PAL, phenylalanine ammonia lyase; C4H, cinnamate 4-hydroxylase; 4CL, 4-coumarate coenzyme A ligase; CHS, chalcone synthase; CHI, chalcone isomerase; F3H, flavanone 3-hydroxylase; F3’H: flavonoid 3’-hydroxylase; DFR, dihydroflavonol 4-reductase; ANS, anthocyanidin synthase; UFGT, (UDP)-glucose:flavonoid 3-O-glucosyltransferase; FLS, flavonol synthase; LAR, leucoanthocyanidin reductase; ANR, anthocyanidin reductase. The mean (black dot) and the standard deviation (vertical black line) are represented in each range-plot for RF and WF. Significantly different means are identified by different letters (tukey HSD, p < 0.05).

The minor anthocyanin of the fruit was quantified as equivalent of cyanidin-3-O-arabinoside (**Table 1**). Indeed, an UHPLC-HRMS analysis of the extract confirmed the molecular formula C20H19O10, indicating the presence of a cyanidin pentoside. Three different cyanidin pentosides have been reported in apple: cyanidin-3-O-arabinoside, cyanidin-3-O-xyloside and cyanidin-7-O-arabinoside, the first one being the most cited (Slatnar et al., 2014; Kokalj et al., 2019). Unfortunately, the retention time does not match and the quantification in equivalent was chosen given that cyanidin-3-O-arabinoside standard was co-injected. WF accumulated significantly more dihydrochalcones and hydroxycinnamic acids with an average concentration of 495.1 and 20755.6 µg/g of dry weight (DW), respectively, against 287.6 and 9603.8 µg/g of DW in RF (**Figure 5**; **Supplementary Table S8**). Data on flavonols were more balanced, nonetheless, RF contents were higher (64.2 µg/g of DW) in comparison to WF (52.6 µg/g of DW). RF accumulated significantly more flavan-3-ols and anthocyanins with an average concentration of 761.1 and 484.7 µg/g of DW, respectively, compared to 586.8 and 37.7 µg/g of DW in WF (**Figure 5**).

Additionally, pH measurements were also carried out on RF and WF. RF showed lower pH values with a mean of 2.97 against 3.11 for WF (**Supplementary Table S5**).

### 3.4 Analysis of differentially expressed genes between RF and WF by RNA-seq and GO-functional analyses

**Figure 6:**
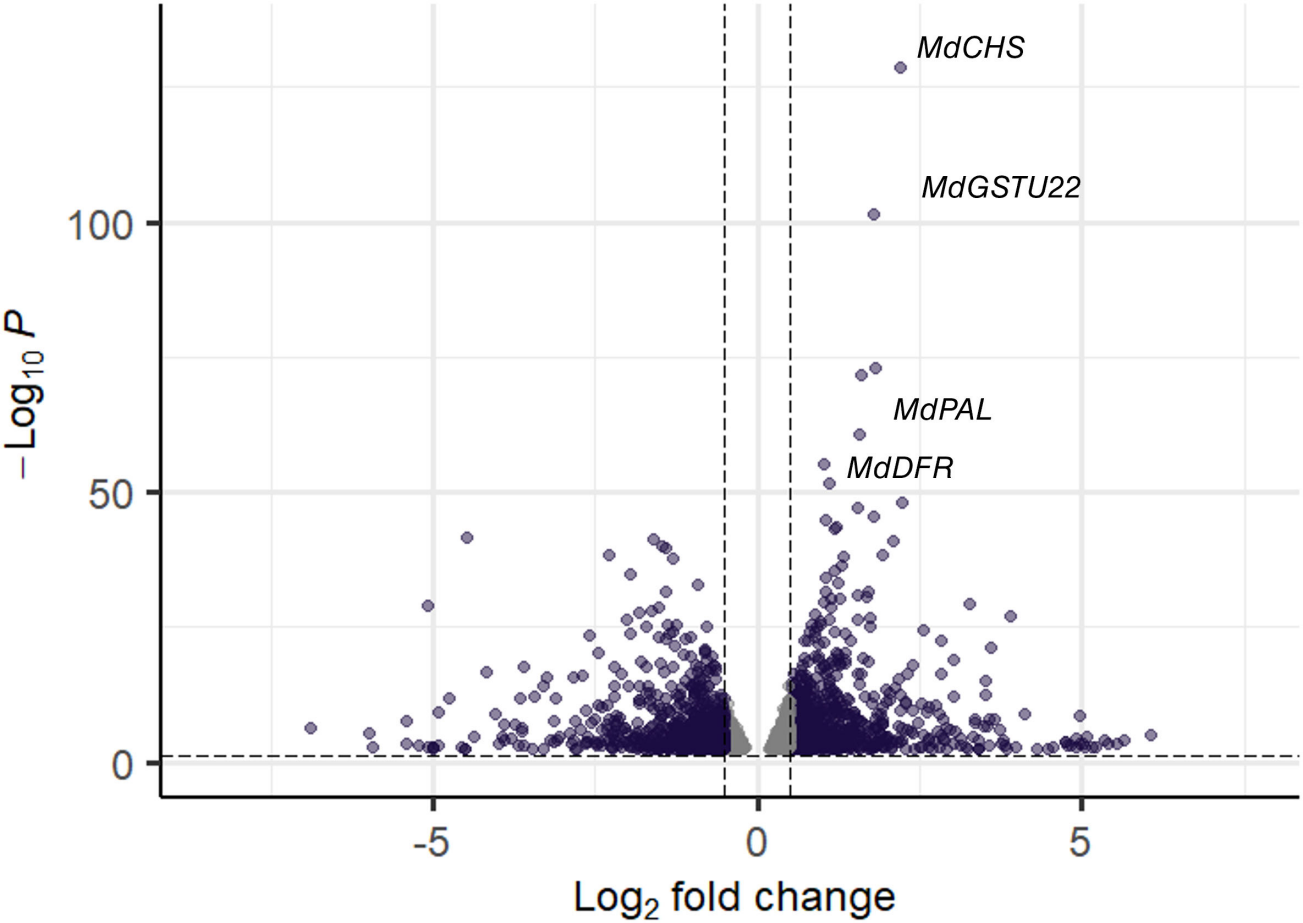
Volcano plot of differentially expressed genes (DEGs) between RF and WF The negative log of p-value (base 10) is plotted on the Y-axis, and the log of the FC (base 2) is plotted on the X-axis. The purple points on this graph represent genes that are significantly differentially expressed between red-flesh (RF) and white-flesh (WF). A total of 2,727 DEGs were identified using a false discovery rate (BH) < 0.05. A total of 853 genes were up-regulated (log Fold Change logFC > 0.5) in RF and 718 genes were down-regulated (logFC < −0.5) in RF compared to WF. The gray points represent genes that are not differentially expressed with log FC is *>* |0.5| and BH *<* 0.05.

To functionally characterize flesh pigmentation pattern, we performed metabolomic and transcriptomic comparisons between RF and WF (**Figures 5**, **6**). The transition step between fully red fruits and heterogeneously pigmented fruit were targeted during fruit development (84 Days After Full Bloom - DAFB). A total of 2,727 DEGs were identified using a false discovery rate (BH) < 0.05. A total of 853 genes were up-regulated (log Fold Change logFC > 0.5) in RF and 718 genes were down-regulated (logFC < −0.5) in RF compared to WF. Among the anthocyanin-related genes, *MdGSTU22* (MD17G1272100), *MdCHS* (MD04G1003300), *MdPAL* (MD04G1096200) and *MdDFR* (MD15G1024100) had the highest differential expression between RF and WF sections (**Figure 6**). This analysis included three semi-biological replicates of RF and WF (fruits from different trees) that are fully reproducible (**Supplementary Figure S1**).

To explore the biological processes of DEGs, we classified the gene ontology (GO) function and analyzed the enrichment (**Supplementary Figure S2**). GO terms were divided into three functional categories: molecular function, cellular component and biological process. DEGs up-regulated in RF were enriched in GO terms associated with response to environmental stimuli (‘response to light stimulus’, ‘response to cold’, ‘response to temperature stimulus’, ‘response to UV’) and flavonoid biosynthetic process (‘regulation of anthocyanin biosynthetic process’, ‘flavonoid biosynthetic process’, ‘anthocyanin-containing compound metabolic process’). DEGs up-regulated in WF were enriched in GO terms associated with hormonal signal transduction (‘response to gibberellin’, ‘response to brassinosteroid’) and flavonoid biosynthetic process (‘flavonoid biosynthetic process’, ‘flavone synthase activity’).

### 3.5 Expression of anthocyanin biosynthethic genes

**Figure 7:**
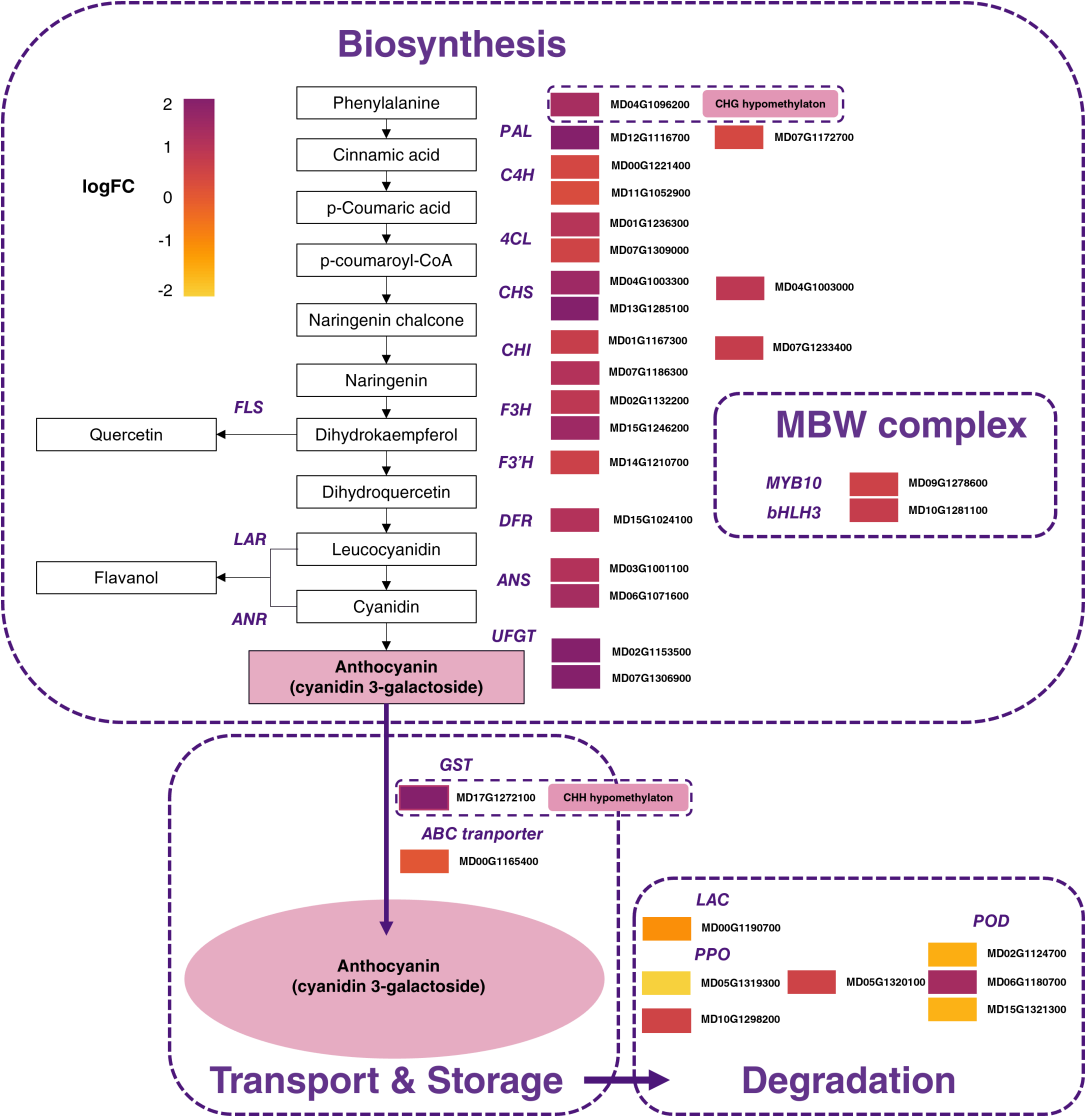
Expression pattern of genes involved in anthocyanin biosynthesis, degradation, and transport pathway between red-flesh and white-flesh. Color boxes from left to right represent values of log Fold Change. PAL, phenylalanine ammonia lyase; 4CL, 4-coumarate coenzyme A ligase; C4H, cinnamate 4-hydroxylase; CHS, chalcone synthase; CHI, chalcone isomerase; F3H, flavanone 3-hydroxylase; F3’H: flavonoid 3’-hydroxylase; DFR, dihydroflavonol 4-reductase; ANS, anthocyanidin synthase; UFGT, (UDP)-glucose:flavonoid 3-O-glucosyltransferase; FLS, flavonol synthase; LAR, leucoanthocyanidin reductase; ANR, anthocyanidin reductase; GST, glutathione S-transferase; ABC, ATP-binding cassette; LAC, laccase; PPO, polyphenol oxidases; POD, class III peroxidases. Color gradient, ranging from yellow to purple represents low, middle, and high values of differential gene expression.

To further investigate transcriptional differences between RF and WF, DEGs encoding TFs related to MBW complex, early anthocyanin biosynthesis gene (EABG) and late anthocyanin biosynthesis gene (LABG) were analyzed. A total of 32 DEGs related to anthocyanin biosynthesis were up-regulated in RF compared to WF with logFC > 0.5 (**Figure 7**).

Globally, genes related to anthocyanin biosynthesis were up-regulated while genes related to phenolic compounds and anthocyanin catabolism were down-regulated in RF compared to WF (**Figure 7**).

### 3.6 Differential methylation analysis Between RF and WF

To explore a potential cause of the observed differential RF-WF gene expression, DNA methylation analysis was performed on the same tissues as those used for the RNAseq analysis. DNA methylation may have an important regulatory effect on gene expression (Zhang et al., 2018). Methylome analysis of RF and WF (**Supplementary Figure S3**) permitted the identification of 1,320 differentially methylated regions (DMRs) in the three contexts (CG, CHG, and CHH), comprising 270 hypomethylated and 1,050 hypermethylated regions in RF compared to WF. Concomitantly, 73 DMRs were associated with DEGs and 2 were found in the proximity of genes involved in anthocyanin synthesis: *MdGSTU22* (MD17G1272100) and *MdPAL1* (MD04G1096200), with a difference in the percentage of methylation of -32.3% and -34.8% respectively. The DMR (CHH context) associated to *MdGSTU22* was located 1,862 bp downstream of the 3’ UTR of the gene. The DMR (CHG context) associated to *MdPAL1* was located at 1,514 bp upstream of the 5’ UTR of the gene.

## 4 Discussion

### 4.1 Genetic control of fruit flesh pigmentation pattern

The genetic determinism of anthocyanin biosynthesis in apple is well characterized and is mainly regulated by the MBW complex through *MdMYB10* activity (Honda and Moriya, 2018; Wang and Chen, 2021). Recently, new regulators of anthocyanin biosynthesis have been identified (Liu et al., 2021), but less is known about spatial regulation of anthocyanin deposition in the fruit flesh. Here we have shown that color patterning of apple red flesh is under genetic control. Indeed, fruit flesh pigmentation patterns were found heritable over three years (2021-2023) with a heritability value of 0.56, thus enabling QTL detection. A QTL for BLUP pigmentation pattern was identified on LG08 and colocalized with two genes that encoded MYB TFs. One R2R3-MYB TF is localized upstream of the QTL mode in the LG8-QTL region and annotated as *MdMYB44* (approximately 23 cM). One uncharacterized MYB-3R colocalized with the LG8-QTL mode (approximately 30.6 cM).

It has been proposed that R2R3-MYB evolved from MYB-3R after the loss of the R1 repeat sequence (Feng et al., 2017). MYB dual activity are well known in anthocyanin synthesis repression (LaFountain and Yuan, 2021) and some R2R3-MYB anthocyanin repressors have been identified in *Malus domestica* (Wang and Chen, 2021). MYB repressors may inhibit the MBW complex via the competition with MYB activators for bHLH binding sites (Yan et al., 2021). Interestingly, *MdMYB44* has been linked to color fading in crabapple leaves by downregulating ABGs (Meng et al., 2022). Recent work suggests that *MdMYB44* negatively regulates vacuolar acidification and decreases malate content (Jia et al., 2021). Vacuolar pH is determinant in anthocyanin color and stability (Zhang et al., 2014). pH variations within apple flesh could lead to anthocyanin-related color loss and differential patterning. Allelic variations in R2R3-MYB sequences are often associated with important phenotypic variations (Espley et al., 2009; Castillejo et al., 2020; Jia et al., 2021) and allelic variations in a new MYB anthocyanin-related activator or repressor could determine formation of pigmentation pattern in red-flesh apple.

Genetic factors other than the genes involved in the MBW complex can regulate anthocyanin biosynthesis (Wang and Chen, 2021). *MdWRKY11* increased the accumulation of flavonoids and anthocyanin in RF apple by promoting LABGs (F3H, FLS, DFR, ANS, and UFGT genes) expression (Wang et al., 2018). However, *MdWRKY11* does not interact with the MBW complex but interacts with *MdHY5* and promotes its activity (Liu et al., 2019). Indeed, the photoresponse factor *MdHY5* was shown to promote anthocyanin accumulation by regulating the expression of the *MdMYB10* gene and downstream ABGs (An et al., 2017). As a consequence, allelic variations associated with *MdWRKY11* and *MdHY5* could be involved in flesh pigmentation patterning.

### 4.2 Developmental cues in pattern acquisition

Whether red-flesh fruits are homogeneously or heterogeneously pigmented at harvest, they are fully red after fruit set and pattern formation only occurs during fruit development. The transition step between fully pigmented fruits and heterogeneously pigmented fruit is determinant in spatial anthocyanin deposition. Wide-targeted metabolite analysis identified differential accumulation in phenolic compounds between RF and WF. As expected, anthocyanins were 15-fold more accumulated in RF than in WF. This drastic difference coincided with increased gene expression of ABGs and MBW complex. Interestingly, some ohnologous genes (Lallemand et al., 2023) exhibited similar differential expression patterns between RF and WF.

The regulatory genes *MdMYB10* and *MdbHLH3* were up-regulated in RF compared to WF (**Figure 7**). MYB-TFs anthocyanin activators are able to bind directly to the promoter of the key gene for anthocyanin biosynthesis and therefore, affect the final anthocyanin accumulation (Yan et al., 2021). As a consequence, positive regulation by MYB is generally associated with a global up-regulation of ABGs (Espley et al., 2012). An additional level of anthocyanin regulation involves WD40 proteins, formerly identified as TTG1 in *A. thaliana* (Gonzalez et al., 2007; Brueggemann et al., 2010), which interact with bHLH through the MBW complex (An et al., 2012). *MdTTG1* (MD01G1228700) was expressed through WF and RF but did not show differential expression (logFC = -0.26). This suggests a required interplay between WD40, MYB-TF and bHLH to upregulate anthocyanin synthesis. In addition to *MdMYB10*, other MYB-TFs were differentially expressed between WF and RF. MYB3 is a transcriptional repressor involved in repression of phenylpropanoid and anthocyanin biosynthesis in *A. thaliana* (Zhou et al., 2017; Kim et al., 2022). DEGs analysis identified a *MdMYB3* (MD15G1411200), down-regulated in RF (logFC = -1.9), making it a potential anthocyanin repressor in red-flesh apple. *MdMYB9* is a positive regulator of anthocyanin and proanthocyanidin biosynthesis in red-flesh apple through the HY5-miR858-MYB9 loop (Li et al., 2023) and jasmonate-mediated signal (An et al., 2021). Subsequent functional analyses demonstrated that *MdMYB9* bound to the promoter regions of flavonoid biosynthetic genes and induced anthocyanin accumulation in calli in the presence of JA (Honda and Moriya, 2018). Unexpectedly, *MdMYB9* was down-regulated in RF (logFC = -0.7, BH = 0.007) while *MdHY5* was not differentially expressed between WF and RF, challenging the current MYB9 related regulatory mechanism. Nonetheless, DEGs related to jasmonate were over-represented in RF, which support the pivotal role of jasmonate signaling in HY5-miR858-MYB9 loop involved in anthocyanin regulation. Plant hormones can regulate anthocyanin biosynthesis through different pathways (Shi et al., 2023). GO-enrichment analysis showed that ‘response to auxin’ related DEGs were over-represented in RF and WF. Interestingly, DEGs related to brassinosteroid and gibberelin were over-represented in WF while DEGs related to jasmonate were over-represented in RF (**Supplementary Figure S2**). Further studies based on hormone measurements, miRNAs and gene expression analysis during fruit development kinetics could help to identify new regulators, mediated by different signals, of anthocyanin biosynthesis. Interestingly, flavan-3-ols and flavonols were also higher in RF (**Figure 5**). Flavonols are derivates of dihydroflavonols synthesized by flavonol synthase (FLS) and flavan-3-ols are synthesized from leuco/anthocyanidins by LAR and ANR. *MdANR* (MD01G1077800, MD05G1335600), *MdLAR* (MD06G1211400, MD13G1046900, MD16G1048500) and *MdFLS* (MD08G1168600, MD15G1353800) were not differentially expressed between RF and WF (**Supplementary Table S6**). Inversely, dihydrochalcones and hydroxycinnamic acids were more abundant in WF than in RF (2-fold). Differential accumulation of early phenolic compounds could be associated with remobilization of flavonoid metabolism or with products resulting from anthocyanin catabolism given that enzymes related to anthocyanin catabolism such as, laccases (LAC), polyphenol oxydases (PPO) and peroxydases (POD), were more expressed in RF compared to WF. Moreover, anthocyanins’ color and stability depend on vacuolar pH via their pH-dependent forms (Zhang et al., 2014). RF showed lower pH values (pH = 2.97) than WF (pH = 3.11), therefore contributing to anthocyanin degradation in WF.

The translocation of glycosylated anthocyanins into the vacuole constitutes a limiting step in anthocyanin accumulation (Buhrman et al., 2022). GSTs are involved in anthocyanin transport by conjugating to ABC transporters (Buhrman et al., 2022). A previous study identified *MdGSTU12* as being involved in apple anthocyanin accumulation (Zhao et al., 2021b). Among the genes with high expression levels (**Figure 6**), *MdGGSTU22* was up-regulated in RF (logFC = 2.2) compared to WF suggesting that this gene is related to anthocyanin accumulation in RF. A recent study (Eichenberger et al., 2023), showed that arGST plays a catalytic role in anthocyanin biosynthesis. However, we did not observe flavonoid substrate competition between RF and WF. Further functional validation is required to understand *MdGSTU22* implication in anthocyanin biosynthesis.

Among the 1,320 DMRs (**Supplementary Figure S3**), we identified a DMR associated with *MdGSTU22* (-32.3% of methylation) as observed in red-flesh kiwi-fruit (Zhang et al., 2023). This DMR (CHH context) was located 1,862 bp downstream of the 3’ UTR of the gene. Promoter regions of genes are generally located in the 5’ region. However, it happens that cis-regulatory elements may also be located in the 3’ region and affect gene expression (Bernardes and Menossi, 2020). Since the function of those 3’ regulatory region in apple are not well-known, a DMR in this region might affect the expression of this particular gene. One ABG (*MdPAL1*) at the start of flavonoid pathway was also linked to a DMR (-34.8% of methylation). These two hypomethylated DMRs were associated with DEGs up-regulated in RF compared to WF, suggesting that DNA methylation could modify anthocyanin-related genes expression as observed in apple skin (Telias et al., 2011; Jiang et al., 2019; Xu et al., 2024). While pigmentation is mainly attuned to developmental process, pigment production and accumulation is also dependent on environmental stimuli (especially light and temperature) (Espley and Jaakola, 2023). GO-enrichment analysis confirmed that RF was more dependent to environmental stimuli than WF with enrichment in GO term related to light (‘response to UV’, ‘response to light stimulus’) and temperature (‘response to cold’, ‘response to temperature stimuli’). Further investigations should be conducted to estimate the genotype, environmental stimuli and hormone related signals effects (Shi et al., 2023), and their interaction, on fruit flesh pigmentation patterning.

### 4.3 Color fading drives pigmentation heterogeneity

QTL analysis revealed for the first time a new locus associated with the genetic determinism of red-flesh pigmentation pattern in apple. The fact that we did not identify a DEG that colocalized within the QTL region could be explained by the experimental setup where we targeted one developmental stage (transition stage between fully red fruits and heterogeneously pigmented fruits) while other activator/repressor of anthocyanin biosynthesis could also be expressed before. QTL may contain master regulators that can control the expression of many genes (Shen et al., 2022). Moreover, PVE of this QTL was 20.2% which suggests that other genetic factors could be involved in pigmentation pattern. This study identified two potential mechanisms involved in pigmentation patterning in red-flesh apple: an activator-repressor system, which may be genetically inherited, as suggested by QTL detection, and color fading, which can occur during fruit development. These mechanisms are intricated, and likely influenced by developmental and environmental factors. Broader QTL analyses with progenies that segregate between homogeneous and heterogeneous pigmented phenotypes, or Genome Wide Association Studies (GWAS) could help to fine mapping LG8-QTL, and improve our understanding of the genetic determinism associated with this original phenotypic trait.

Altogether, our results support that WF result from color fading while maintenance through development of RF is associated with an increased expression of anthocyanin-related positive regulators. Indeed, while ABGs and MBW complexes were up-regulated in RF, a lack of anthocyanin transport (down-regulation of *MdGSTU22* in WF compared to RF) and an increase in phenolic catabolism (up-regulation of *MdLAC*, *MdPPO* and *MdPOD* in WF compared to RF) were concomitant with the color fading process in WF. Color fading has been identified in apple organs such as flower (Han et al., 2020), leaves (Meng et al., 2022) and fruit skin (Xu et al., 2024). Here, color fading was linked to variations in anthocyanin related genes expression while methylation levels could modulate inner pigmentation pattern. Extension of comparative study on other genotypes could enable the identification of new anthocyanin regulatory mechanisms involved in anthocyanin spatial deposition and therefore flesh pigmentation pattern.

## Data Availability

The datasets presented in this study can be found in online repositories. The names of the repository/repositories and accession number(s) can be found in the article/**Supplementary Material**. GEO accession numbers (online repositories) : GSE269807 and GSE269214.

## Supplementary material

Supplementary Figure 1 Heatmap-cluster analysis of differentially expressed genes (DEGs) between RF and WF The standardized amount of expression is represented by different colors. GO-term and enrichment analysis of DEGs.

Supplementary Figure 2 GO-enrichment analysis of differentially expressed genes (DEGs) between RF and WF GO terms were divided into three functional categories: molecular function, cellular component, and biological process. The Y-axis represents the number of DEGs enriched in each GO term.

Supplementary Figure 3 Analysis of differentially methylated regions (DMRs) between RF and WF **(A)** Number of DMRs among the three contexts (CG, CHH, CHG). **(B)** Distribution of percentage of methylation for DMRs among the three contexts. **(C)** Position of DMRs associated with MdGSTU22 and MdPAL1.
