# Supplementary figures and images for: Fade into you: genetic control of pigmentation patterns in red-flesh apple (Malus domestica)
Source: Front Plant Sci. 2025 Jan 13;15:1462545. doi: 10.3389/fpls.2024.1462545 (PMC11770013; doi:10.3389/fpls.2024.1462545)

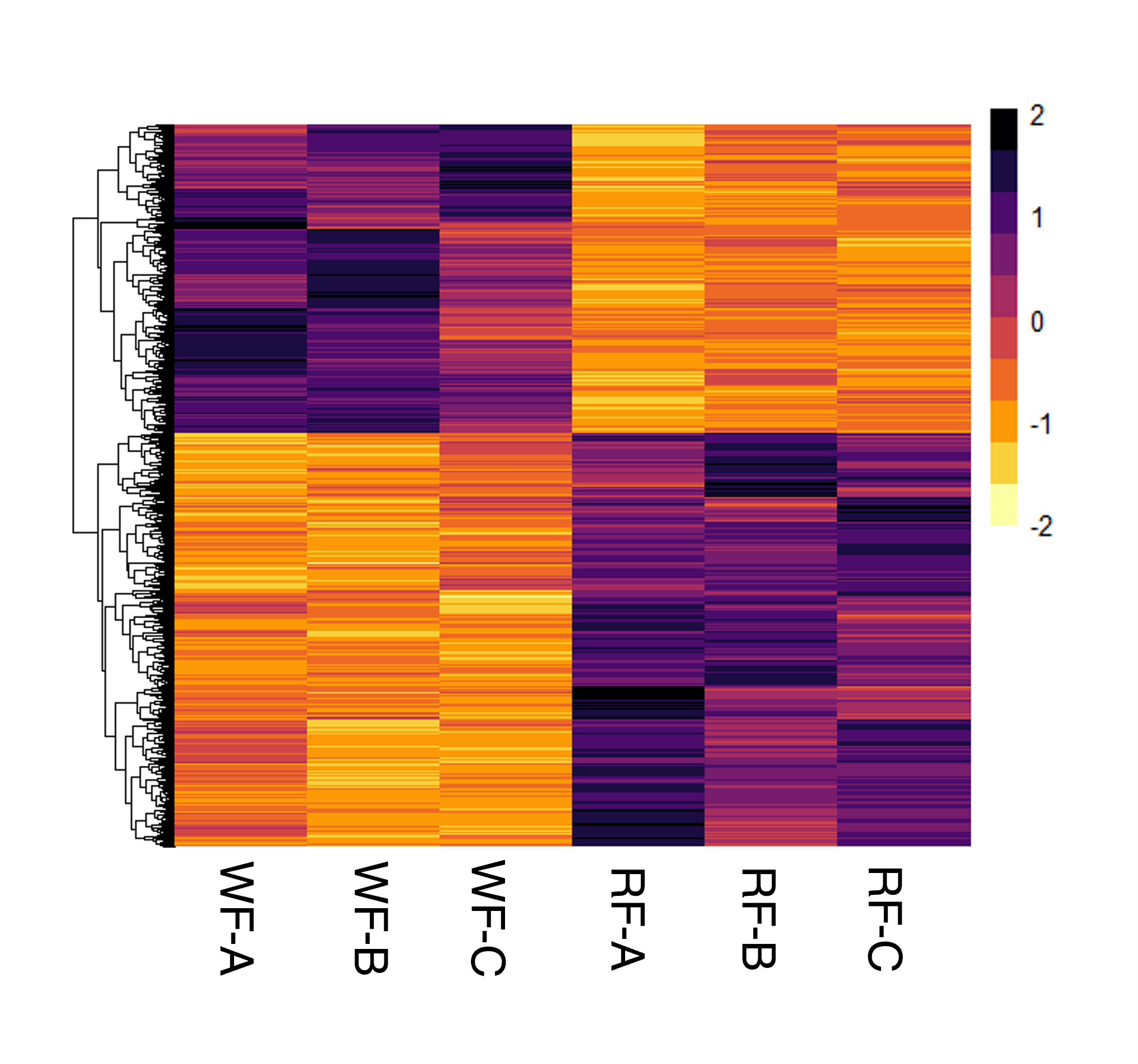

Supplement: Supplementary Figure 1 — Heatmap-cluster analysis of differentially expressed genes (DEGs) between RF and WF The standardized amount of expression is represented by different colors. GO-term and enrichment analysis of DEGs. [file Image1.tif]

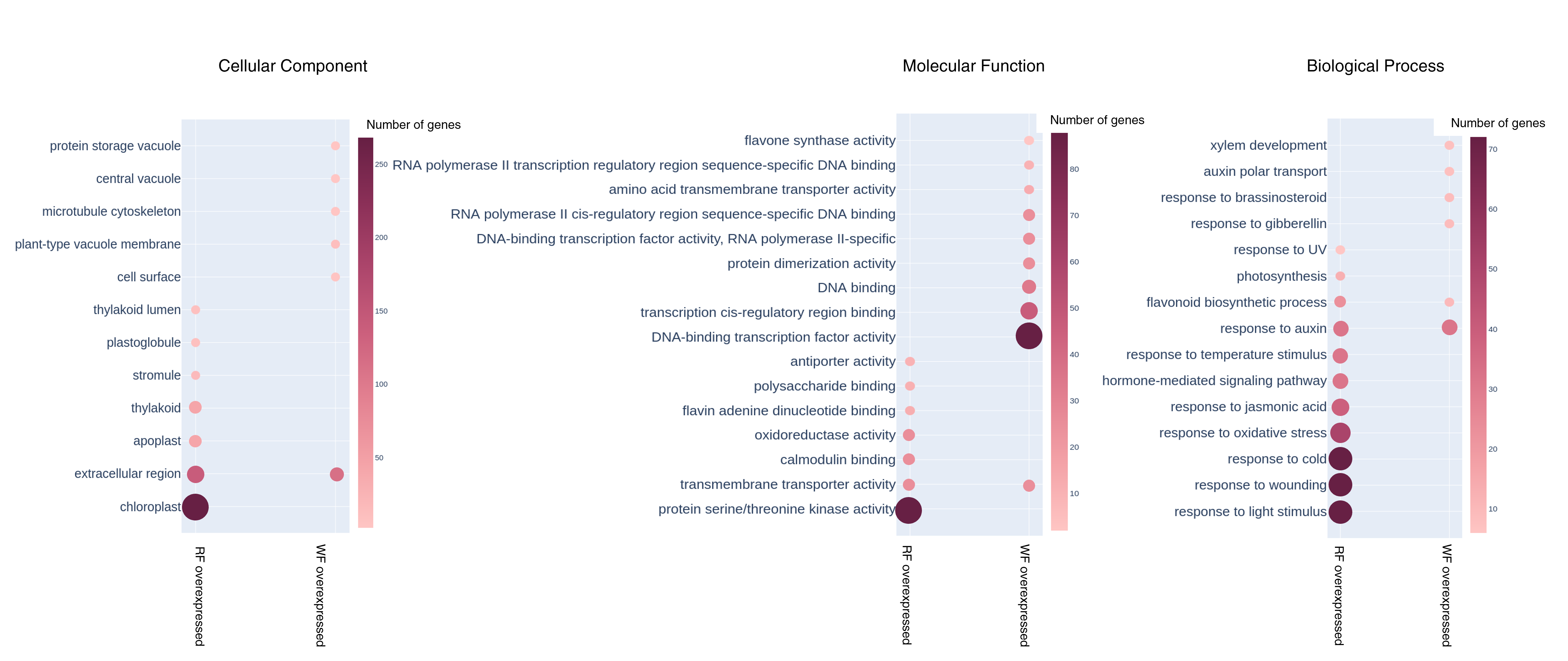

Supplement: Supplementary Figure 2 — GO-enrichment analysis of differentially expressed genes (DEGs) between RF and WF GO terms were divided into three functional categories: molecular function, cellular component, and biological process. The Y-axis represents the number of DEGs enriched in each GO term. [file Image2.tif]

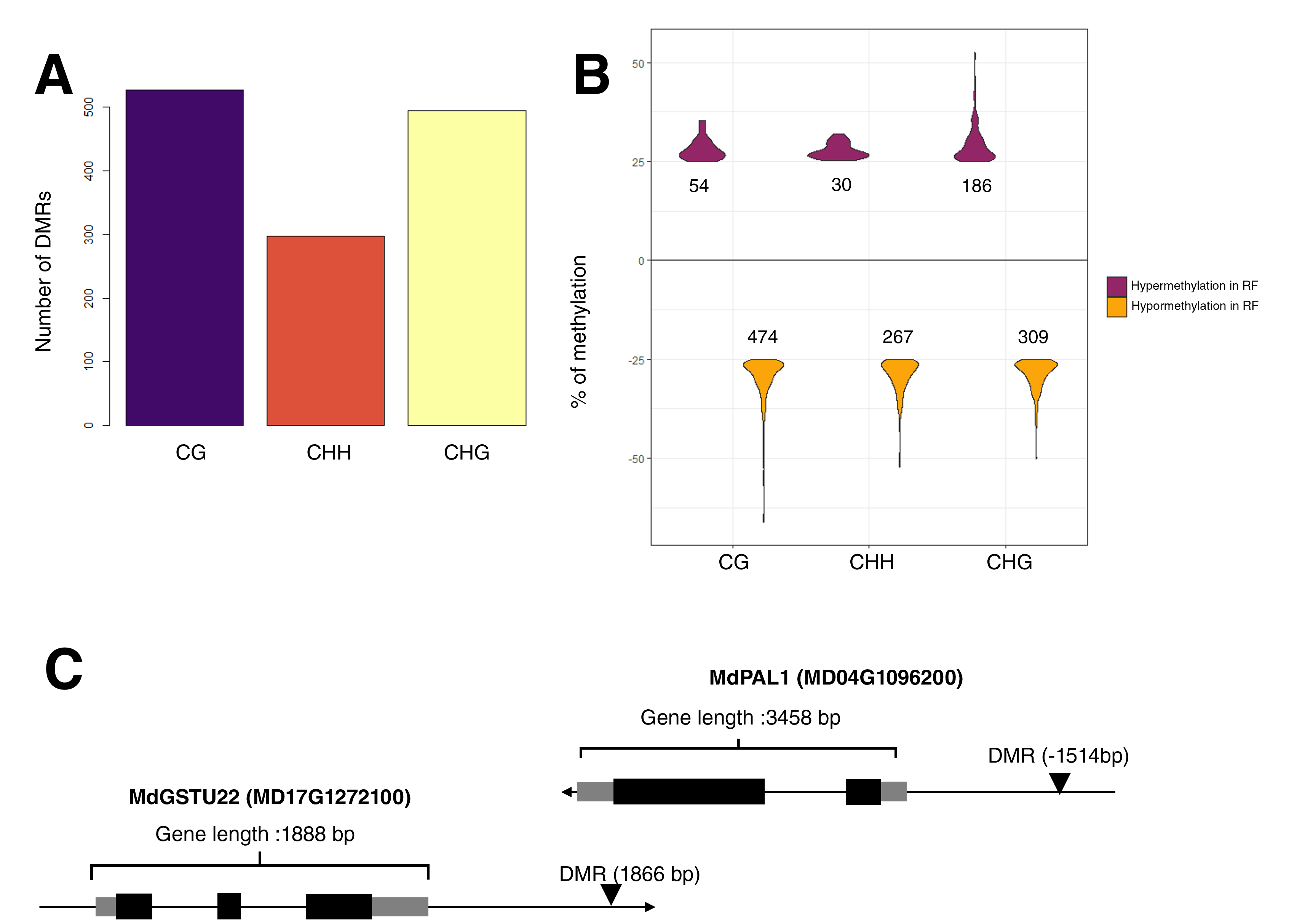

Supplement: Supplementary Figure 3 — Analysis of differentially methylated regions (DMRs) between RF and WF (A) Number of DMRs among the three contexts (CG, CHH, CHG). (B) Distribution of percentage of methylation for DMRs among the three contexts. (C) Position of DMRs associated with MdGSTU22 and MdPAL1. [file Image3.tif]
